# The role of the general practitioner in managing age-related hearing loss: perspectives of general practitioners, patients and practice staff

**DOI:** 10.1186/s12875-020-01157-2

**Published:** 2020-05-14

**Authors:** Rebecca J. Bennett, Susan Fletcher, Nicole Conway, Caitlin Barr

**Affiliations:** 1 Department of Audiology and Speech Pathology, University of Melbourne, Parkville, VIC 3010 Australia; 2 Ear Science Institute Australia, Suite 1, Level 2, 1 Salvado Road, Subiaco, WA 6008 Australia; 3 Department of General Practice, University of Melbourne, Parkville, VIC 3010 Australia; 4 Better Hearing Australia (VIC), Suite 1, Level 2/517 St Kilda Rd, Melbourne, VIC 3004 Australia

**Keywords:** General practice, General practitioner, Practice nurse, Practice manager, Hearing, Hearing loss, Auditory, Audiology, Ear, Concept mapping

## Abstract

**Background:**

For people with hearing loss, the General Practitioner (GP) can play an instrumental role in early detection of hearing loss as well as guiding appropriate and timely choices for addressing hearing concerns. The aim of this study was to generate a conceptual framework for understanding the role of the GP in managing age-related hearing loss.

**Methods:**

Concept mapping techniques were used to gather the perspectives of GPs (*n* = 8), adults with hearing loss (*n* = 22), and professionals working with GPs (*n* = 5), in Australia. Participants generated statements describing the role of the GP in managing age-related hearing loss, and then grouped the statements to identify key themes, via an online portal.

**Results:**

Ninety-eight items describing the role of the GP in managing age-related hearing loss were identified across six concepts: 1) Determine - Diagnose - Discuss, 2) Ask - Assess - Act, 3) Know - Refer - Coordinate, 4) Inform - Advise - Partner, 5) Educate - Strategise - Encourage, 6) Reassure - Support - Empower.

**Conclusions:**

The role of the GP in managing age-related hearing loss is multifaceted and requires partnership that motivates and empowers patients’ to overcome their hearing concerns. Enlisting the help of Practice Nurses, Practice Managers and local audiologists could help GPs improve their hearing loss detection and intervention rates.

## Background

Hearing connects us with our environment, providing an awareness of our surroundings, warning for potential dangers, and an avenue for social connection. However, the sense of hearing can be taken for granted, with hearing loss often viewed as an unfortunate but benign side effect of aging. The notion of “age appropriate hearing loss” disregards the devastating impacts that unmanaged hearing loss can have on the many facets of an individual’s life [1, 2]. Hearing loss can compromise the ability to communicate, and consequently impacts on social network size, [3] engagement in meaningful conversations, [4] participation in social activities, [5, 6] and mental health [7]. Despite advances and benefits of hearing-related technologies (such as hearing aids and hearing implants), [8] help-seeking for these technologies remains low, [9, 10] with adults delaying help-seeking for hearing loss by an average of 8.9 years [11]. Consequently, millions of adults continue to experience functional and psychosocial impacts due to hearing loss.

Research has highlighted the influential role of support from others on hearing help-seeking, including significant others (family and friends) [12–14] as well as health professionals [15, 16]. However, while the General Practitioner (GP) is often the first point of contact for people seeking advice or referral for hearing health concerns, [16] research suggests that screening for hearing loss and the provision of timely and appropriate referrals are not routine practice in the primary care setting [16, 17]. Research suggests that only a minority of suitable patients are being considered for hearing screening, with up to 85% of older patients reporting having received no spontaneous advice from their GP regarding hearing [18]. Furthermore, data suggests that even patient-directed enquiries are sometimes dismissed by GPs [18]. In a population-based cohort study in Australia, less than half of those seeking some form of help from their GP actually received referral for treatment or support services for their hearing loss [16]. Reasons for this include time constraints, insufficient training regarding hearing screening and treatment, and the need for GPs to focus on more pressing medical issues [19, 20].

For people with hearing loss, the GP can play an instrumental role in guiding appropriate and timely choices for addressing hearing concerns. A population-based consumer survey in the USA reported that people with hearing loss are five times more likely to seek a hearing solution when their GP gives a positive recommendation for hearing healthcare [15]. The real-world benefits of GP-facilitated hearing intervention were demonstrated in the UK, where introduction of hearing screening questionnaires reviewed by the GP during clinical consultations identified previously undetected cases of hearing loss in 26% of participants; 58% of whom obtained hearing aids [21]. Six months later, 87% of those fitted with hearing aids were regularly using them, and 79% self-reported a reduction in hearing handicap (based on the Hearing Handicap Inventory for the Elderly Screening survey [22]. Surveys of GP attitudes towards hearing loss in the elderly suggest that GPs have a high level of awareness about their older patients’ susceptibility to hearing loss, but appear less certain about their role in facilitating early intervention, including doubts regarding the effectiveness of hearing aids and negative perceptions about how older patients prioritise hearing [20, 23]. Although GPs are well placed to support people to manage their age-related hearing loss, many appear not to be identifying hearing loss, actively encouraging help-seeking, or appropriately referring patients to specialist services. To better understand current barriers and facilitators to hearing loss identification and management in primary care, this study explored the perceived role of the GP in managing age-related hearing loss from the perspectives of GPs, patients with hearing loss, and professionals working alongside GPs.

## Methods

This study used concept mapping techniques to develop a framework for understanding the perceived role of the GP in managing age-related hearing loss. Concept mapping is a semi-qualitative approach wherein participants put forward statements that described their perceptions or experiences related to a topic of interest, [24] in this case the provision of hearing healthcare support in the general practice setting. Participants then group the statements to identify key themes. Group concept mapping is a well-established technique used in applied health services research, [25] including audiology research [26–28].

### Participants

Three stakeholder groups were identified and recruited for this study: GPs currently working in Australia; adults with hearing loss who had sought help from a hearing clinic; and professionals who provide training for or work with GPs in Australia, including but not limited to Practice Managers, Practice Nurses, and University lecturers. GPs were recruited via the Victorian Primary Care Practice-based Research Network (VicReN [29]), the Royal Australian College of General Practitioners (RACGP), and through professional networks. Adults with hearing loss, who were under the care of a GP in Australia, were recruited through two audiology clinics (Melbourne and Perth, Australia), and two community hearing organisations**.** Professionals that provide training to and/or work with GPs were recruited via GPs involved in this study, through the Australian Primary Healthcare Nurses Australia (APNA), and through the Australian Association of Practice Management (AAPM).

### Procedure

This project was conducted under the oversight of the University of Melbourne Human Research Ethics Committee, 1,851,579.2.

Participants contributed to two online data collection sessions using Concept Systems Inc. software [30]: one Brainstorming and one Grouping session.

#### Brainstorming

Participants were asked to provide statements in response to the open-ended question “What should the role of the GP be in managing age-related hearing loss?”, and were provided with the prompt “In my opinion, the role and responsibility of GPs in managing age-related hearing loss is…”. Participants were encouraged to brainstorm as many responses as possible, entering them into the online system. All statements were available for other participants to see so that they could build on each other’s ideas. Participants were not able to change or comment directly on others’ statements, only contribute new statements. Participants were not identifiable to each other. The brainstorming session was accessible for 6 weeks, after which the research team reviewed all of the responses to eliminate those that were irrelevant (not strictly answering the research question), and edited the remainder for clarity where required. This final list of statements was then used for the grouping session.

#### Grouping

Approximately 2 weeks after completion of the brainstorming session, participants were asked to log into the portal again to complete the grouping session. On this occasion they were presented with the list of edited statements and asked to group them in a way that made sense to them. Participants were provided with the following instructions: (a) there was no right or wrong way to group the statements, (b) a statement could be put in its own group if it was unrelated to the other statements or if it stood alone as a unique statement, and (c) they should not have a “Miscellaneous” or “Other” group [27, 31]. Participants were instructed to provide a short title that described the content of each group they had created.

#### Data analysis

Data analysis was conducted using Concept Systems software [30] and IBM SPSS Statistics. Multidimensional scaling was used to generate a point map graphically displaying the results of the grouping task [31]. Each statement was represented by an individual point on the point map, with the proximity of the points indicating the relationship of statements to one another. That is, points that appeared closer together on the point map had been grouped together more often by the participants than points that appeared further apart from each other. The strength of the multidimensional scaling analysis was tested by computing a stress index value, with a value below 0.365 indicating an acceptable fit [32]. Hierarchical cluster analysis was used to determine the overarching concepts through generation of cluster maps, graphically depicting clusters of points (statements) based on a consensus of how the participants grouped the individual statements [31]. All four researchers met to discuss and decide upon the appropriate number of clusters by reviewing the cluster configurations and discussing which of the cluster maps most appropriately represented the data, that is, which configuration of clusters most made sense given the statements contained within its clusters [27, 33]. These decisions were also informed by bridging scores, indicating how often participants grouped the statements in this way [27]. Each cluster, containing a unique concept, was then given a name that represented the concept contained therein, and informed by the names put forward by the participants during the grouping session.

A split-half reliability measure was conducted to evaluate the validity of the final concept map, applying the Spearman-Brown Prophecy Formula correction [31]. A correlation above 0.7 was considered high, [34]. indicating an accurate representation of the participants’ grouping data by the concept map.

## Results

Thirty-five participants completed the brainstorming activity: eight GPs; 22 adults with hearing loss; and five professionals working with GPs. This sample meets the recommendations of at least 20 people for a concept mapping brainstorming activity [31]. Participants ranged in age from 28 to 86 years (Median 61, SD 18.16), 24 were female (68.6%) and 11 were male (31.4%). Nineteen participants completed the sorting activity (86.4% retention rate), meeting the minimum of 15 participants as recommended by the literature [35]. Of these, three were GPs, 11 were adults with hearing loss, and five were professionals working with GPs. These participants ranged in age from 28 to 86 years (Median 67, SD 19.68), 14 were female (73.7%) and five were male (26.3%). The sorting activity was more complex and time consuming (30–45 min) than the brainstorming activity (10–15 min) to complete. Three participants completed the sorting activity with phone support from the research team, two opted out of this task because they found the task too complex, and one indicated that they were overseas and unable to complete this part of the study.

During the brainstorming activity four participants expressed opinions that the GP does not have a role in managing age-related hearing loss, in that people liaise directly with an audiologist for their hearing services and do not involve their GP. Statements of this effect were excluded from the study as they did not directly answer the research question “What should the role of the GP be in managing age-related hearing loss?”

Editing the remaining raw brainstormed statements resulted in a final list of 98 statements used in the grouping task. The individual statements described both biomedical and person-centred approaches to patient care. Biomedical actions included detection, diagnosis, treatment and referral. Person-centred actions included listening, supporting, motivating and reassuring.

**Fig. 1:**
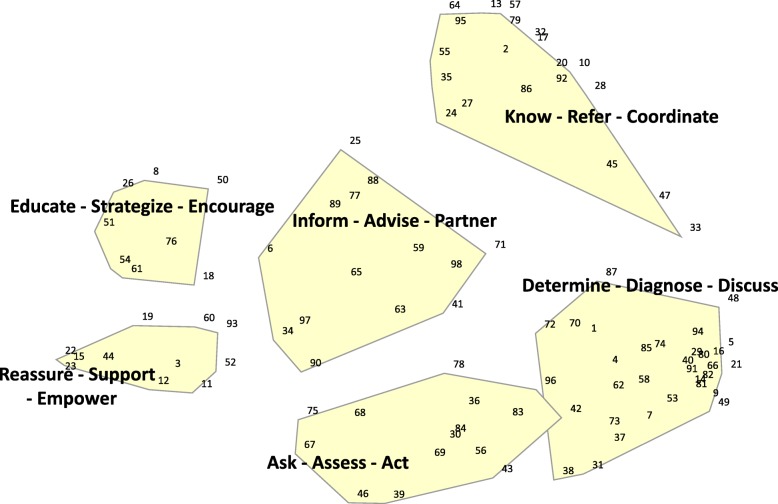
Key concepts that describe the role of the GPs in managing age-related hearing loss

The number of groups created by participants ranged from four to 21 (mean 8.63, SD 4.53). The research team (RB, CB, SF & NC) determined that the six-cluster configuration most appropriately represented the participants’ brainstorming and grouping data. This six-cluster concept map (Fig. 1) had a stress index of 0.2359, suggesting acceptable fit to the data [32]. Reliability testing of the grouping task using split-half correlation and application of the Spearman–Brown correction resulted in a reliability estimate of 0.885, suggesting high consistency in the way individual participants grouped the data [24]. A description of the six clusters and the statements contained within each can be found in Table 1.

**Table 1:** Six concepts describing the role of the GP in managing age related hearing loss

Concept	Description	Statements (bridging score)
Determine - Diagnose - Discuss	Clinical processes of evaluating hearing, determining the cause of hearing loss, and discussing the diagnosis with the patient.	29. Ensure a basic hearing assessment is conducted as part of age-related assessments (0.00) 80. Conduct such tests as may be possible with equipment generally available in GP surgeries (0.01) 16. Conduct some basic tests to determine extent of any issues (0.03) 82. Examine for ear canal and ear drum problems (0.03) 40. Have access to a basic Audiology Tool to assist diagnosis and management of hearing loss (0.03) 53. Identify hearing impairment (0.04) 81. Examine to ensure no physical cause, such as ear wax (0.04) 14. Check for physical causes of hearing loss, such as wax build up, ear drum health, etc. (0.04) 74. Routine assessment of hearing when performing +75yo health check (0.05) 66. Assess hearing as part of the test linked to maintaining a driver’s licence (0.05) 85. Screen for hearing loss annually (0.05) 91. Rule out sinister (not age-related) hearing loss (0.05) 21. Provide a diagnosis (0.06) 58. Implement a hearing questionnaire or similar at +75yo health check (0.07) 9. Conduct ENT examination to rule out things like wax or structural causes (0.08) 5. Investigate hearing problems (0.10) 7. Pick up hearing loss in patients (0.10) 94. Provide diagnosis (0.10) 73. Identify patients who may be suffering from a hearing impairment (0.11) 4. Recognise hearing problems (0.12) 49. Measure hearing levels at ALL health assessments, i.e. +75yo health assessments, 45-49yo Health Assessments and all Annual Health Checks (0.13) 37. Ask if the patient is having hearing difficulties (0.14) 62. Perform wax removal (0.15) 31. Enquire about and/or notice potential hearing loss particularly with at-risk patients (0.20) 70. Recommend regular hearing checks (0.22) 38. Open the discussion about hearing with people entering the age period when they are at-risk of hearing loss (0.24) 1. Provide help managing other issues related to hearing, such as wax issues, middle ear issues, or infections (0.24) 72. Ask whether the patient is managing their hearing. Many people get hearing checks at local audiology clinics and don’t bother talking to the GP about it. GPs need to ask these questions if they want to be informed of their patients’ health and health-related activities (0.27) 48. Have access to an Audiology Tool to measure hearing levels (0.33) 87. Recommend hearing assessment, promoting early intervention (0.43)
Ask – Assess - Act	Actively look for signs and symptoms of hearing loss, assess functional hearing and impact on daily activities, normalise discussions on hearing rehabilitation, and promote action. Actively make hearing loss part of the conversation, and be the catalyst for change.	42. Assist with early detection and intervention, as people have much better physical and social outcomes the earlier they receive treatment (0.16) 96. Assess for risks of untreated hearing loss, e.g. higher risk of falls (0.22) 83. Assess whether elderly patients hear and comprehend what is discussed in their consultation (0.24) 78. Be aware of the signs of hearing loss, such as asking for repeats, and patient reports of withdrawal from social situations (0.25) 43. Open the discussion on hearing loss (0.27) 30. Keep a closer eye on patients as they age, as the impacts of untreated hearing loss can be significant (0.30) 69. Talk about hearing loss with patients as they age. Discuss hearing loss each step of the way (0.31) 56. Ask the patient whether they have any concerns regarding there hearing (0.33) 84. Assist to help normalise the issue and suggest getting hearing checked (0.35) 36. Involve the patient in any procedures that they (the GP) may be conducting (0.36) 68. Get involved. GPs don’t currently raise hearing loss, but they should (0.38) 39. Normalise discussion about hearing loss and ensure it is on the radar for all adults over 60 years (0.49) 75. Advise patients on the importance of monitoring hearing (0.53) 67. Discuss the importance of hearing well to be safe when driving (0.60) 46. Especially in rural areas, it is essential that GPs and Practice Nurses are educated and enabled to do basic audiology checks and educate people (1.00)
Know - Refer - Coordinate	Refer patients for ear- and hearing-related services, and have knowledge of local specialists to assist making appropriate recommendations.	79. Refer patient to appropriate professional, such as audiologist or ENT (0.01) 57. Provide referral to specialists for tinnitus (0.01) 13. Coordinate audiology appointments (0.05) 17. Refer to specialist services as needed (0.09) 32. Just refer on to a reputable, knowledgeable hearing service that is not biased or specialised in one brand product only (0.13) 95. Arrange audiology testing and hearing aids (0.15) 92. Be a referral access point (0.17) 20. Provide referrals to hearing specialists (0.17) 64. Know the appropriate referral pathways for ear related disease. Patients should be referred to Audiology before ENT for hearing- and balance-related issues (0.18) 2. Assist the patient in accessing resources and/or services to optimise their hearing (0.19) 10. Refer patients to audiology (0.26) 28. Simply refer on to a hearing service/specialist (0.32) 86. Have knowledge of ear diseases and who to refer to for these specific problems (0.36) 55. Encourage use of audiology services (0.37) 27. Promote audiology services to improve the patient’s communication skills (0.42) 24. Provide information on available paths of care: ENTs, audiologists, psychologists, etc. (0.42) 35. Be informed on which hearing aid providers are acting ethically, not simply pushing the most expensive hearing aids (0.43) 33. Make sure the patient gets the most appropriate testing done as soon as possible (0.53) 47. Encourage patients to have full audiology testing, as early detection is key to better outcomes (0.66) 45. Work closely with their practice nurses to ensure they are providing hearing assistance (0.72)
Inform – Advise - Partner	Be an active part of the patient’s hearing rehabilitation, including understanding their personal journey, checking in to see how they are going, and interacting with their hearing healthcare team.	41. Have a good understanding of hearing loss (0.32) 63. Discuss balance issues (0.36) 98. Assist with management options (0.38) 97. Discuss prevention of further hearing loss (0.38) 65. Understand balance disorders, and have the skills to test and treat BPPV (a specific type of balance disorder, often easily managed with exercises) (0.39) 34. Fully explain the sensations experienced with tinnitus and vertigo, as they can be debilitating (0.41) 90. Listen to hearing concerns (0.41) 6. Advise and encourage patients to wear their hearing aids (0.45) 77. Check in with patients who have recently gotten hearing aids, to see how they are going with them and report any problems back to audiologist (0.46) 71. Recommend hearing aids, if needed (0.47) 88. Read correspondence from audiologists, and discuss with the patient. Acknowledge that they are trialling new hearing aids and ask how they are going (0.47) 89. Acknowledge that the patient has recently obtained hearing aids, and provide support, e.g. ask whether they are receiving benefit and recommend they discuss any concerns with their audiologist (0.49) 59. Provide support to those who need further assessment (0.50) 25. Be an informed “gatekeeper” for what is needed next (0.57)
Educate – Strategise - Encourage	Educating patients on the importance of managing hearing health, including intervention options available. Providing personalised practical support, including communication strategies and encouragement to seek specialist help.	54. Provide education (0.41) 61. Educate patients about the importance of hearing health as part of healthy ageing (0.43) 18. Provide some simple strategies to minimise the impact of the hearing loss (0.46) 51. Educate and encourage patients about the benefits of properly fitted hearing aids (0.49) 76. Advise patients on the importance of intervention for hearing loss (0.51) 8. Provide hearing loss education (0.63) 50. Educate and encourage patients about audiology services (0.66) 26. Give a person confidence to seek further audiology testing (0.75)
Reassure - Support - Empower	Being sympathetic to patient concerns, understanding the impacts of hearing loss and providing emotional support.	23. Be supportive and provide positive guidance (0.32) 22. Provide re-assurance that hearing loss isn’t “the end” of things (0.32) 15. Reassure patients if they’re anxious about the hearing rehabilitation process (0.33) 52. Provide trusted advice (0.40) 93. Provide support regarding the impact of hearing loss on other areas of the patient’s health (0.43) 60. Understand the impact of hearing loss on all areas of mental and physical health (0.45) 3. Understand that hearing is closely related to quality of life (0.45) 19. Provide strategies to cope with hearing loss (0.46) 44. Help the patient realise that while hearing loss is a normal part of ageing there are things can be done to help (0.48) 12. Explore the effects of hearing loss on quality of life (0.50) 11. Explore the impact of the hearing loss on health, e.g. depression (0.56)

NB. The statement bridging scores indicate which statements were anchors in a specific area of the map and which ones were bridging across different areas of the map. A lower bridging score indicates that participants more often grouped this statement in this concept. Thus the statements with the lower bridging scores most represent the core meaning of the concept

The six concepts identified were:

1. *Determine - Diagnose - Discuss*: Clinical processes of evaluating hearing, determining the cause of hearing loss, and discussing the diagnosis with the patient.
2. *Ask - Assess - Act*: Actively look for signs and symptoms of hearing loss, assess functional hearing and impact on daily activities, normalise discussions on hearing rehabilitation, and promote action. Actively make hearing loss part of the conversation and be the catalyst for change. Statements in this concept described the need for the GP to actively keep hearing loss front of mind.
3. *Know - Refer - Coordinate*: Refer patients for ear- and hearing-related services and have knowledge of local specialists to assist making appropriate recommendations. Statements contained within this concept described both the process of making referrals, and also the benefits of knowing local professionals and making referrals based on personal knowledge of the referral pathway. Statements within this concept emphasised the importance of referring to hearing healthcare professional who were knowledgeable, independent, and provided personalised services.
4. *Inform - Advise - Partner*: Be an active part of the patient’s hearing rehabilitation, including understanding their personal journey, checking in to see how they are going, and interacting with their hearing healthcare team.
5. *Educate - Strategise - Encourage*: Educating patients on the importance of managing hearing health, including intervention options available. Providing personalised practical support, including communication strategies and encouragement to seek specialist help. Statements contained within this concept described information pertinent to hearing aids as well as preventing further hearing loss deterioration.
6. *Reassure - Support - Empower*: Being sympathetic to patient concerns, understanding the impacts of hearing loss and providing emotional support.

The two concepts *Determine - Diagnose - Discuss* and *Ask - Assess - Act* overlapped, suggesting that the concepts were interrelated. Statements #96 *“Assess for risks of untreated hearing loss, e.g. higher risk of falls”* and #42 *“Assist with early detection and intervention, as people have much better physical and social outcomes the earlier they receive treatment”* were positioned close to the boarder of these two concepts, suggesting that participants did not always agree on the grouping of these statements (potentially due to their double-barrelled nature).

## Discussion

The purpose of this study was to investigate the perceived role of the GP in managing age-related hearing loss, from the perspective of GPs, adults with hearing loss, and professionals working with GPs. At a time when GPs are considered not just medical specialists, but specialist in life, it is unsurprising that GPs are perceived to play a role in the biomedical and psychosocial aspects of hearing loss.

Hearing loss is described as an invisible disability, as people do not often recognise hearing loss in themselves. As a result, many adults live for years, or even decades with the psychosocial impacts of unmanaged hearing loss [11]. Participants in the current study described the role of the GP in facilitating early detection of hearing loss. Statements put forward by participants described the need for GPs to detect the subtle signs of hearing loss, raise their concerns, normalise conversations about hearing loss, and monitor patients as they age. Although previous research has called for GPs to improve their hearing loss detection and intervention rates, [16, 18] there does not appear to be a simple solution for this. GPs are responsible for detecting the widest range of conditions of any speciality. To cover 75% of the conditions they manage, GPs need to have knowledge of more than 100 problems [36]. Furthermore, GPs will manage on average 1.5 health problems in each consultation [37]. They are time poor, and often need to prioritise perceived more pressing health conditions. Given the invisibility of hearing loss, and the unlikeliness that patients will spontaneously raise their hearing concerns with the GP, it is no wonder that GPs do not routinely detect hearing loss in their patients. However, given their frequent contact with patients at risk for hearing loss (older adults and those with co-morbid health conditions), GPs are well placed to support people to recognise and manage their age-related hearing loss. Targeted interventions are needed to help GPs to improve their rates of detection and intervention for hearing loss.

Participants in this study recommended the incorporation of routine hearing screening programs in general practice. Implementing hearing screening programs targeting older adults has been shown to increase the detection rate for hearing loss, [21, 38] and subsequently increase the number of patients receiving hearing loss intervention, including hearing aids [39]. In a trial of a hearing screening program in the Netherlands, hearing loss was detected in 57% of general practice patients screened [40]. This was new information to the GP in 54% of cases and resulted in GPs recommending intervention in 84% of cases. Similarly, a screening program in Austria identified hearing loss in 15% of general practice patients, 23% of which were new cases previously unknown to the GP, and 100% of which were referred for specialist hearing assessment [41]. Participants in the present study recommended that hearing screening programs be incorporated into existing health checks. Routine hearing assessments are already endorsed by the Australian Government through Medicare-funded health checks for adults aged 75 years and older [42]; however, our findings suggest that there is scope to increase the degree to which hearing is addressed as part of these assessments [16].

One potential option to support GPs in prioritising hearing discussions might be the redistribution of tasks within the primary care setting. Over time, the work of the GP has shifted towards the management of chronic disease, and general practice itself has become organised through increasingly complex groups of doctors, Practice Nurses, administrative managers and support staff [43]. Consistent with previous research describing the role of the Practice Nurse in screening for and providing education around hearing loss, [44] two of the statements put forward by participants in the current study described the role of the Practice Nurse in supporting the GP with hearing loss detection and management. Routine hearing screening is not difficult nor time consuming. The various methods currently used in the general practice setting include direct inquiry regarding hearing difficulties, use of questionnaires (such as the Hearing Handicap Inventory for the Elderly [22]), tunning forks, the finger rub test, the whispered voice test, handheld screening devices, and audiometers [45–47]. By incorporating screening for hearing loss among older adults in the workflow of Practice Nurses, some of the negative effects of untreated hearing loss could be prevented or reduced.

Patient-centred care is central to the mission of healthcare, especially self-management of chronic disease [48]. In this context, participants in the present study identified a number of areas in which GPs could *‘partner’* with their patients, for example: developing an understanding of their experience of hearing loss, encouraging help-seeking, providing education and training on hearing loss management (including ear wax management and communication training), promoting compliance with intervention options (including hearing aid use), and working with health professionals to take a multidisciplinary approach to patient care. The rising number of people living with chronic (and multiple) health conditions requires GPs to be patient-focussed, to seek understanding of what individual patients need from health and social services, and to provide tailored support, to reduce dependency and improve quality of life [49, 50]. Emphasis has been placed on the need for GPs to better listen to patients, and improve shared decision making based on patients’ individual priorities and preferences [51]. This need for a patient-centred approach is not unique to people with hearing loss.

The importance of multidisciplinary care and the GP’s role in coordinating this was particularly highlighted through the *Know - Refer - Coordinate* concept. The statements contained within this theme described not only the process of referral to specialist audiology services, but the importance of the GP having knowledge of who they are referring to, and to refer to reputable hearing services. A comparable scenario is a 2015 enquiry into mental health management in the primary care setting explored the barriers and facilitators to GP referral for specialist mental health services [52]. Improving relationships between GPs and allied health practitioners was identified as the most effective strategy to address barriers to successful referrals. Requesting and providing feedback (monitoring quality) and delivering training and/or resources were equally identified as the second most effective strategy to address barriers to referral. However, given that GPs do not necessarily have the time to build relationships with potential referrers, thus there may be a role for audiologists to play regarding the upskilling of local GPs with regards to hearing loss detection, discussion and referral. In rural and remote locations, a lack of local audiology and/or ENT services presents as an additional barrier to GPs developing referral networks, [53] and despite an increase in tele-audiology services [54] additional support and upskilling for GPs in these underserved areas may be required.

The *Know - Refer - Coordinate* concept also described the importance for the GP to have an understanding of the “next steps”, and to assist and encourage the patient through the referral process. The notion of ‘warm’ referral has started to emerge in the medical literature and practice [55]. Traditional ‘cold’ referral involves providing the patient with information about another agency or service. This places onus on the patient to actively follow-up and seek help from the recommended service, and also requires the patient to retell their story and effectively communicate their needs. Alternatively, a ‘warm’ referral sees the GP contact the agency or service on the patient’s behalf and explain patient’s circumstances and the reason for referral. This may occur via telephone, scheduling a group meeting, or writing a report or case history. The additional time commitment that warm referrals can involve means they are not always feasible for GPs. However, there may be opportunities for other practice staff to facilitate warm referrals or for the development of system improvements to assist.

### Limitations & future directions

Participants self-selected for the study, and although the sample sizes were sufficient for concept mapping procedures, data from only 35 participants may have biased the content of the statements captured. Furthermore, participants described their experiences within the Australian context of service delivery, potentially biasing the findings. Future research could also explore the experience of non-help-seekers to better understand the barriers to seeking support for hearing loss in the General Practice setting.

## Conclusions

The perceived role of the GP in managing age-related hearing loss is multifaceted and includes detection and diagnosis, discussion and empowerment, as well as referral and ongoing support. For adults with hearing loss, the GP can play an instrumental role in the early identification of hearing loss, guiding appropriate and timely choices for addressing hearing concerns, and providing partnership that motivates and empowers patients’ to overcome their hearing concerns, and the psychosocial impacts that develop due to untreated hearing loss. There is scope to optimise how this occurs in GP clinical practice.

## Data Availability

All brainstorming data generated and analysed during this study are included in this published article. The participant grouping data are not publicly available but are available from the corresponding author on reasonable request.

## Abbreviation

GP: General Practitioner
